# A multi-centre randomized controlled trial comparing electrothermal arthroscopic capsulorrhaphy versus open inferior capsular shift for patients with shoulder instability: Protocol implementation and interim performance: Lessons learned from conducting a multi-centre RCT [ISRCTN68224911; NCT00251160]

**DOI:** 10.1186/1745-6215-7-4

**Published:** 2006-02-02

**Authors:** NG Mohtadi, RM Hollinshead, PJ Ceponis, DS Chan, GH Fick

**Affiliations:** 1 Sport Medicine Centre and Division of Orthopaedic Surgery, University of Calgary, 2500 University Drive NW, Calgary, Alberta, T2N 1N4, Canada

## Abstract

**Background:**

The shoulder is the most frequently dislocated joint in the body. Multiple causes and pathologies account for the various types of shoulder instability. Multi-directional instability (MDI) and multi-directional laxity with antero-inferior instability (MDL-AII) are similar in pathology, less common and more difficult to treat. These instabilities are caused by ligamentous capsular redundancy. When non-operative management fails for these patients, quality of life is significantly impaired and surgical treatment is required to tighten the ligaments and joint capsule. The current reference (gold) standard treatment for MDI/MDL-AII is an open inferior capsular shift (ICS) surgical procedure. An alternative treatment involves arthroscopic thermal shrinkage of redundant capsular tissue to tighten the joint. However, there is a lack of scientific evidence to support the use of this technique called, electrothermal arthroscopic capsulorrhaphy (ETAC). This trial will compare the effectiveness of ETAC to open ICS in patients with MDI and MDL-AII, using patient-based quality of life outcome assessments.

**Methods:**

This study is a multi-centre randomized clinical trial with a calculated sample size of 58 patients (p = 0.05, 80% power). Eligible patients are clinically diagnosed with MDI or MDL-AII and have failed standardized non-operative management. A diagnostic shoulder arthroscopy is performed to confirm eligibility, followed by intra-operative randomization to the ETAC or ICS surgical procedure. The primary outcome is the disease-specific quality of life questionnaire (Western Ontario Shoulder Instability Index), measured at baseline, 3, 6, 12 and 24 months. Secondary outcomes include shoulder-specific measures (American Shoulder and Elbow Surgeons Score and Constant Score). Other outcomes include recurrent instability, complications and operative time.

The outcome measurements will be compared on an intention-to-treat basis, using two-sample independent t-tests to assess statistical significance. A Generalized Estimated Equations (GEE) analysis will determine whether there is an effect over time.

**Discussion:**

This ongoing trial has encountered unexpected operational and practical issues, including slow patient enrollment due to high intra-operative exclusion rates. However, the authors have a greater understanding of multi-directional laxity in the shoulder and anticipate the results of this trial will provide the medical community with the best scientific clinical evidence on the efficacy of ETAC compared to open ICS.

## Background

The shoulder is the most frequently dislocated joint in the body with an estimated annual incidence of 1–2% [1,2]. Shoulder instability affects people in all decades of life, most commonly in the late teens to mid thirties, when people are most active, both recreationally and vocationally. The disability and time loss from work, as well as the effect on quality of life represent a significant clinical problem for the population and the healthcare system.

'Shoulder instability' is a generic and somewhat vague term encompassing a spectrum of disorders, from a painful shoulder with occult subluxation to frank dislocation. It can be further classified according to the direction of the instability (anterior, posterior or multi-directional), the etiology (traumatic vs. atraumatic), the duration of the problem (acute vs. chronic or recurrent) and whether it occurs involuntarily or under the patient's own volition [3].

The pathology of shoulder instability has been well delineated in the literature. Bankart originally described the 'essential lesion' of recurrent post-traumatic anterior shoulder instability as capsulolabral avulsion off the anterior-inferior glenoid rim [4,5]. Others have since elucidated the importance of ligaments intrinsic to the shoulder capsule in maintaining stability of the joint. In particular, during abduction and external rotation of the upper arm, the anterior band of the inferior glenohumeral ligament (IGHL) is placed under tension and restrains anterior translation of the humeral head [6,7]. The Bankart lesion ('essential lesion') represents a detachment of the IGHL's insertion or anchor, limiting its ability to develop tension and thus allowing for abnormal translation [8]. From a biomechanical standpoint an analogous situation occurs if the ligament, while retaining its anchors, becomes stretched, attenuated or redundant as a result of repetitive minor trauma or inherent laxity [9,10]. Indeed, this is the main pathological lesion in patients with multi-directional instability (MDI) and multi-directional laxity with antero-inferior instability (MDL-AII). Both of these groups of patients have ligamentous or capsular redundancy as the primary cause of their instability and present with similar clinical findings.

The initial approach for treatment of MDI and MDL-AII involves non-surgical rehabilitation, including modification of activities, strengthening of the shoulder musculature and proprioceptive training [11,12]. Should rehabilitation fail, open and arthroscopic surgical interventions can be considered. The open Inferior Capsular Shift (ICS) procedure is the current reference standard treatment for ligamentous or capsular redundancy [13,14]. Open ICS involves incising, overlapping and suturing the capsule, resulting in less capsular redundancy.

In recent years, attention has been focused on arthroscopic surgical treatment for all types of shoulder instability in an attempt to decrease surgical morbidity and minimize loss in range of motion [15-19]. The results of arthroscopic procedures have been previously reported to be less successful compared to those of equivalent open surgical procedures in patients with the same type of instability [15-18,20] because of the inability to address capsular ligamentous redundancy [21-24]. Although the arthroscopic suture plication approach minimizes the damage to the surrounding musculature, it is less effective for addressing capsular redundancy [25,26].

One existing method to address capsular redundancy is laser capsulorrhaphy, or laser assisted capsular shrinkage (LACS), which can be performed arthroscopically, but is costly and delivers high bursts of energy that can be difficult to control [27,28]. In addition, some failures due to "rebound" ligament stretching, technical difficulties, and laser replacement and maintenance costs remain significant concerns. An alternative to laser technology is radio-frequency energy, which has been used for various applications in medicine [29-32]. Electrothermal Arthroscopic Capsulorrhaphy (ETAC) is a more recent procedure whereby a small radio-frequency probe, or "heat probe" [29], is inserted through an arthroscopic portal to deliver heat to the capsular ligamentous tissue. This method achieves temperatures necessary to denature collagen, shrinking redundant ligamentous tissue in the shoulder in a controlled manner, [29-32] thus reducing excess capsular volume. The ETAC tec hnology allows for more controlled energy delivery, and the ability to monitor the temperature generated at the probe-tissue interface. Not only is ETAC less costly and technically easier to perform than laser technology, it is also less invasive than open ICS, since it preserves muscle attachments and is less likely to compromise the proprioceptive control of the shoulder [33]. However, it is unclear whether these purported benefits of ETAC can improve shoulder instability and patient quality of life in the population of patients with MDI and MDL-AII.

Technology, rather than scientific clinical evidence, is often the driving force in orthopaedic surgery today. Within the context of shoulder instability, this trend has been evident with the advent of arthroscopic techniques, and specifically with the introduction of ETAC. While this may be a disturbing statement to most surgeons, there are only five published randomized clinical trials related to shoulder stabilization surgery [34-38], none of which have addressed this new technology or the patient population with shoulder instability due to capsular redundancy. Recent publications on the thermal treatment of shoulder instability are limited to retrospective case series [39-43], with one prospective consecutive series of patients comparing laser assisted capsular shrinkage (LACS) to radio-frequency capsular shrinkage (ETAC) [44]. These publications do not lend themselves to a meta-analytic approach because of inconsistent definitions of patient populations, combined surgical techniques, variation and lack of validated outcomes, and no comparative groups. There has been little corroborative basic science or clinical evidence to support ETAC procedures. This dearth of scientific evidence is in grave contrast to the numbers of procedures performed. A variety of authors have repeatedly supported this position [45-51], emphasizing the need for an appropriate trial that is subsequently evaluated in the context of peer-reviewed research. Thus, this multi-centre study is designed to compare the theoretical advantages and effectiveness of the ETAC procedure to the open ICS procedure in the context of a randomized clinical trial.

## Methods

### Design

This Canadian study is designed as a national multi-centre randomized clinical trial with all surgical collaborators being fellowship-trained shoulder surgeons. The protocol and informed consent process have been approved by the University of Calgary Conjoint Health Research Ethics Board (ID: 10650). In addition, approval has been granted by the institutional ethics board of each participating centre in the trial.

The primary objective is to determine if there is a difference in disease-specific quality of life outcome over 2 years in MDI and MDL-AII patients undergoing open ICS or ETAC surgery, as measured by the Western Ontario Shoulder Instability (WOSI) Index [52]. Secondary objectives include determination of: a) the recurrence rate of instability (post-operative symptomatic subluxation or dislocation); b) the difference in overall shoulder functional status, as measured by standardized shoulder joint-specific outcome assessments (American Shoulder and Elbow Surgeons (ASES) Score [53], and Constant Score [54]); and c) the difference in surgical time between the two treatment groups.

#### Outcome measures

The primary outcome used to compare the treatment groups is the Western Ontario Shoulder Instability (WOSI) Index, a self-administered, disease-specific outcome designed to measure quality of life in patients with shoulder instability [52]. The WOSI index has 21 questions, divided into four categories (physical symptoms, sport/recreation/work, lifestyle, and emotions), whereby each question is scored on a 100-mm visual analogue response scale. The responses are measured and summed to provide an overall score out of 2100. A lower score reflects a better quality of life. This index has been demonstrated to be valid, reliable and responsive in a patient population comparable to that of the proposed study [52]. In addition, it is used as the primary outcome in ongoing and published clinical trials investigating traumatic anterior shoulder instability [34,55,56], and laser assisted capsular shift for MDI patients [40]. It should be made clear, that the scoring as originally described has been arithmetically converted for the purposes of this trial to average the scores on each question resulting in a possible score of 0–100, with 100 being the best possible score.

One secondary outcome is the American Shoulder and Elbow Surgeons (ASES) score, a shoulder-specific assessment tool developed by the American Shoulder and Elbow Surgeons Society for use in all types of shoulder problems [57]. It consists of both patient self-assessment and physician assessment components. The patient self-evaluation is divided into two sections: pain and activities of daily living (ADL). Pain is recorded on a visual analogue scale and ADL's are recorded on a numeric scale. The overall score is an equal weight of the two sections and produces a score out of 100. A higher score reflects a better outcome. A score is not calculated in the physician assessment component. This portion is divided into four segments to provide information about range of motion, physical signs, strength and instability.

Another secondary outcome is the Constant Score adopted by the European Shoulder Society for overall clinical functional assessment of the shoulder [54]. The Constant Score records a variety of shoulder measurements, including an objective test of strength using a spring-loaded measuring device. The Constant Score is based on a 100-point scoring system calculated from a self-assessment portion that evaluates pain and ability to perform tasks of daily living, and a clinical assessment portion that tests shoulder strength and active range of motion. A higher score reflects a better outcome.

Additional outcomes include the documentation of recurrent instability, complications and operative time. Recurrent instability is defined as a self-report of one or both of the following criteria: a) A minimum of two subluxation events, where subluxation is defined as the symptomatic translation of the humeral head relative to the glenoid articular surface [58]; and/or, b) At least one re-dislocation, where dislocation is defined as an increased motion of the humeral head relative to the glenoid to the point of complete separation of articular surfaces [58].

The outcome measures are collected at each clinical follow-up appointment in a standardized fashion and by a trained independent assessor who is blinded to treatment status. If patients do not return to the clinic or miss an appointment, all attempts are made to have the questionnaires completed through mail-out or internet-based formats.

#### Randomization

Consenting patients, clinically diagnosed with either MDI or MDL-AII, and who have failed non-operative rehabilitative management, are randomized to either an ETAC or open ICS surgical treatment. Computer-generated (STATA 8.2, StataCorp, USA), stratified block randomization is used to ensure that imbalances between group assignments do not occur during the course of the trial. Stratification is based on two variables: 1) surgeon – to account for any differences between surgeons, and, 2) diagnosis (MDI and MDL-AII) – to account for any differences in the severity of pathology. Thus, the stratification process results in a separate set of sealed numbered opaque envelopes for each participating surgeon and for each type of shoulder instability (MDI or MDL-AII).

Randomization occurs in the operating room following a diagnostic arthroscopy. Once the surgeon confirms that the patient meets the eligibility criteria, the next consecutive envelope is opened by the study research coordinator or the assigned circulating nurse, which randomly allocates the patient to either open ICS or ETAC treatment. If the patient is ineligible, the surgeon performs the most appropriate procedure as he/she sees fit.

### Interventions

#### Open Inferior Capsular Shift (ICS)

The open ICS procedure used in this study was described by Schenk and Brems [11], which is a modification of Neer and Foster's original procedure [13]. A standard deltopectoral approach is utilized. The tendon of the subscapularis is incised 1 cm medial to the lesser tuberosity. The interval between the tendon and capsule is developed, working from lateral to medial, until the capsule is completely exposed. No muscle is left on the capsule. The rotator interval is identified and, if a rotator interval lesion is present, it is closed using number 1 non-absorbable sutures. This is done with the arm adducted and externally rotated 30 degrees. The capsule is incised in a T-shaped fashion with the vertical limb based laterally and the transverse limb placed so as to lie perpendicular to the glenoid and intersect it near its equator. In patients with MDI, the lateral capsule is released antero-superiorly from the rotator interval to the equator posteriorly on the humeral neck. In patients with MDL-AII, the release extends from the rotator interval to the 7 o'clock position (right shoulder) or 5 o'clock position (left shoulder) on the neck of the humerus. This effectively tightens the two bands of the inferior glenohumeral ligaments, the middle glenohumeral ligament and the rotator interval. The bone adjacent to the articular surface on the surgical neck of the humerus is roughened to create a bleeding bony bed. With the arm held in 0 degrees flexion, 30 degrees abduction, 30 degrees external rotation, the inferior leaflet of the capsule is shifted superiorly and slightly laterally and sutured using a non-absorbable suture to the rim of the capsule remaining laterally [59]. The superior leaflet is shifted inferiorly and sutured in a similar fashion. The subscapularis is repaired at its anatomic length using interrupted sutures. The skin is closed in the usual fashion, dressings applied and the shoulder placed in a shoulder immobilizer.

#### Electrothermal Arthroscopic Capsulorrhaphy (ETAC)

The Oratec Vulcan Generator electro-thermal system (Oratec Interventions Inc., Menlo Park, CA, USA) is the energy system utilized for the ETAC arm of the trial. The unit is automatically set to deliver a temperature of 75 degrees Celsius and 40 watts [60]. Following or during diagnostic arthroscopy, an anterior working portal is established just above the superior border of the subscapularis tendon such that access to the inferior recess of the joint is easily attainable. In patients with MDI, the arthroscope is moved to the anterior portal and the heat probe is introduced through the posterior portal. The capsule is shrunk using a grid pattern [61] until excess volume is diminished. The extent of the heat probe application is identical to the landmarks used for the open ICS. Care is taken to avoid applying heat to the capsule in the region from 5–7 o'clock within 1 centimetre of the rim of the glenoid – this is done to avoid the axillary nerve at its most vulnerable point [62]. The current method of heat application utilizes a grid pattern, as determined by recent basic science work [61]. This pattern is less likely to cause dissolution of the capsule and subsequent catastrophic capsular loss, as reported by Weber [63]. The portals are closed in the usual fashion, dressings applied and the shoulder placed in a shoulder immobilizer.

In both surgical arms of the study the duration of the surgical procedure and any intra-operative complications are noted. The patients remain in hospital until pain and nausea control is achieved using standard treatments according to each surgeon. This may include both out-patient and in-patient surgery depending upon the patient and surgeon preferences.

Post-operative immobilization and rehabilitation are identical in both groups.

### Subjects

Eligible subjects are assessed in the clinic and must meet the following inclusion criteria:

a) Age 14 years or greater.

b) Diagnosis of MDI [13] or MDL-AII [64]. Diagnosis will require two or more of the following:

i. symptomatic translation (pain or discomfort) in one or more directions: anterior, inferior and/or posterior.

ii. ability to elicit unwanted glenohumeral translations that reliably reproduce symptoms with one of the following tests: anterior and posterior apprehension tests, the anterior and posterior load and shift tests, the fulcrum test, the relocation test, the Fukuda test and/or the push-pull or stress test with the patient supine [65].

iii. presence of a positive sulcus sign of 1 centimetre or greater gap that reproduces the patient's clinical symptoms of instability and should be both palpable and visible.

iv. symptoms of instability: subluxation or dislocation

c) Informed written consent.

d) Failed at least 6 months of non-operative treatment.

e) Confirmed capsular-ligamentous redundancy as determined by diagnostic arthroscopy examination.

Subjects meeting the following criteria are excluded from the study:

a) Neurologic disorder (e.g. axillary nerve injury; syringomyelia).

b) Cases involving third party compensation.

c) Patients with primary posterior instability.

d) A bony abnormality (Hill Sachs/Bony Bankart) on standard series of x-rays consisting of a minimum of an antero-posterior view, lateral in the scapular plane and an axillary view.

e) Presence of a Bankart lesion on arthroscopic exam of the joint.

f) Presence of an unstable biceps anchor, (e.g. SLAP lesion [66]), on arthroscopic exam of the joint.

g) Presence of a full thickness rotator cuff tear, on arthroscopic exam of the joint.

### Sample Size Calculation

#### Minimal Clinically Important Difference (MCID)

A utility index model [67-69] was utilized by the authors of this trial to determine the MCID for the WOSI questionnaire. First, patients completed the WOSI and were informed of their score converted to an average out of 100. For the purposes of this pilot study, the 100-point WOSI index was reversed such that a score of 100 represented the best possible quality of life score and 0 the worst. Then, using this system, patients were asked: 'What is the smallest increase in your score that you would consider to represent a 'significant improvement'? A pilot study of 15 MDI patients illustrated that an averaged MCID for improved quality of life was 32.7 points (range 20 points to 50 points) with a median score of 30 points (data not shown). Based on the lower end of the range and the median, an MCID of 20 points was chosen. Furthermore, the authors and collaborators of this study met (London, ON, Canada: June, 2001) and collectively agreed on a 20 point (20%) change in the overall score as a clinically important difference, while blinded to the results of the pilot patient project. Therefore, choosing a 20% difference in group means ensures a sufficient sample size to test the alternative hypothesis that the ETAC procedure demonstrates a different outcome from the standard ICS. In addition, the smaller mean difference of 20% was chosen to calculate a more conservative sample size; therefore minimizing the chance of making a Type II error.

#### Sample size

The sample size calculation is based on the existing baseline information from the first thirty patients entered into the trial. The first 30 patients had a mean WOSI score of 1461.68 with a normal distribution of the data and a standard deviation of 364.52. The estimated minimal clinically important difference between treatment groups is 20%.

Sample size parameters:

▪ Comparing 2 independent group means with a MCID between means = 20%

▪ Mean group 1 = 1461.68; Mean group 2 = 1169.34; Standard Deviation (SD) = 364.52

▪ Predicted patient drop-out rate (based on drop-out rate to date) = 12%

Using these sample size parameters, an alpha value p = 0.05, and 80% power, the calculated sample size is 29 patients per group (Table 1).

**Table 1:** Sample size calculation for the required number of subjects in each treatment group (double sided).

**Alpha level("p" value)**	**Power**			
	**60%**	**70%**	**80%**	**90%**

0.10	12	15	20	27
0.05	16	20	25*	33
0.01	25	30	37	47
0.001	40	46	54	65

N per group = 25* Expected dropout 12% 25/.88 = 28.4 Total per group 29 Grand Total **58 patients**

### Blinding

Due to the nature of this surgical trial, the surgeon and the patient are not blinded to the intervention. However, a trained independent assessor blinded to treatment status conducts the follow-up examinations in a standardized fashion. This minimizes potential biases introduced by the examiner when performing the physical assessment and recording the data. To maintain the blinded state at each follow-up appointment, patients wear a t-shirt, rather than a tank top, to conceal surgical scars. The assessor does not have access to the patient chart. To avoid observer bias, the physical examination and the administration of study questionnaires are standardized across centers. Additionally, the surgeon is blinded to the results of the pre-operative WOSI before the patient's eligibility has been determined. This ensures that the eligibility of the patient is not based on subjective characteristics and circumstances.

### Data analyses

The primary analysis will involve a comparison of the mean WOSI scores between the two surgical treatment groups on an intention-to-treat basis. This analysis is a two-sample independent t-test to assess whether there is a statistically significant difference between groups for the mean WOSI score at 2 years. The 5% significance level will be employed. The underlying assumption for the WOSI data is that a normal distribution will exist, but if the sample distribution is determined to depart from normal, then a Wilcoxon rank sum test will be performed. In addition, a Generalized Estimated Equations (GEE) [70] analysis will be conducted to determine whether there is an effect over time (repeated measures) (i.e. 3, 6, 12 months and 2 year follow-ups).

The planned secondary analyses are performed using a 5% significance level. No p-value adjustment is required for multiple outcomes, as treatment effectiveness is based only on the primary variable (i.e. WOSI score). Secondary outcome measurements will be analyzed in the following manner: 1) The ASES scores and Constant Scores using t-tests and GEE analysis in a similar fashion to the primary outcome; 2) Recurrence rates will be compared between treatment groups using a Fisher's exact test; and, 3) Operative times between the two treatment groups will be compared using a logarithmic scale and a t-test.

### Data safety monitoring committee and trial steering committee

The Trial Steering Committee (TSC) has 4 members with backgrounds in epidemiology, clinical trial methodology and orthopaedic surgery. The duties of this committee are to conduct and manage the trial, review recruitment rates and overall compliance with data collection and study protocol.

The Data Safety Monitoring Committee (DSMC) monitors all adverse events and major complications that occur throughout this trial. The Committee is formed by 4 independent members who are neither investigators nor collaborators in this trial. Their backgrounds include orthopaedic surgery, joint injury and arthritis research, sport medicine and epidemiology.

The DSMC provides an advisory role to the Trial Steering Committee and is not involved in conducting or managing the trial. The DSMC meets annually to review recruitment reports and overall compliance with data collection and study protocol, and subsequently provides feedback and recommendations to the TSC.

In the event of a major complication, the DSMC members hold an emergency meeting to review of the operative report and patient chart, and determine whether the complication could occur with greater incidence in one intervention compared to the other. To prevent a biased evaluation, the group is blinded to treatment allocation. The DSMC then provides an immediate recommendation for the TSC to decide whether the event is serious enough to warrant stopping the trial. The TSC will weigh the benefits of the interventions and the risks of potential adverse events to collectively decide on the continuation or discontinuation of the clinical trial.

## Discussion

The introduction of ETAC technology for orthopaedic use was accepted for the treatment of shoulder instability without the clinical evidence to support this procedure. This randomized controlled trial will determine the effectiveness of ETAC compared to the reference standard, open ICS, in reducing capsular redundancy in a highly selected patient population presenting with MDI or MDL-AII, exclusive of additional shoulder pathologies. The completion of this study has been slower than anticipated because of unexpected practical and operational issues addressed below.

### Enrollment is slower than anticipated

The greatest difficulty encountered in this trial to date is with enrolling eligible patients. The calculated sample size for this trial was 58 patients, however only 49 patients have been collectively enrolled from the participating centres between 1999 and 2005. Three reasons can explain these difficulties. Firstly, in designing this trial, each shoulder surgeon belonging to JOINTS Canada (Joint Orthopaedic Initiative for National Trials of the Shoulder) estimated that 10 to 15 patients would present with MDI and MDL-AII in their practice annually and all patients would be referred for surgical treatment. However, these numbers were overestimated by each surgeon. For example, in Calgary, a city of nearly a million people and a catchment population of up to 2 million, only 79 patients over a 5-year period met the clinical criteria of MDI or MDL-AII. Therefore, the annual estimate of 10–15 patients more appropriately reflects the number of MDI and MDL-AII patients seen in a large urban centre, rather than per surgeon.

**Figure 1:**
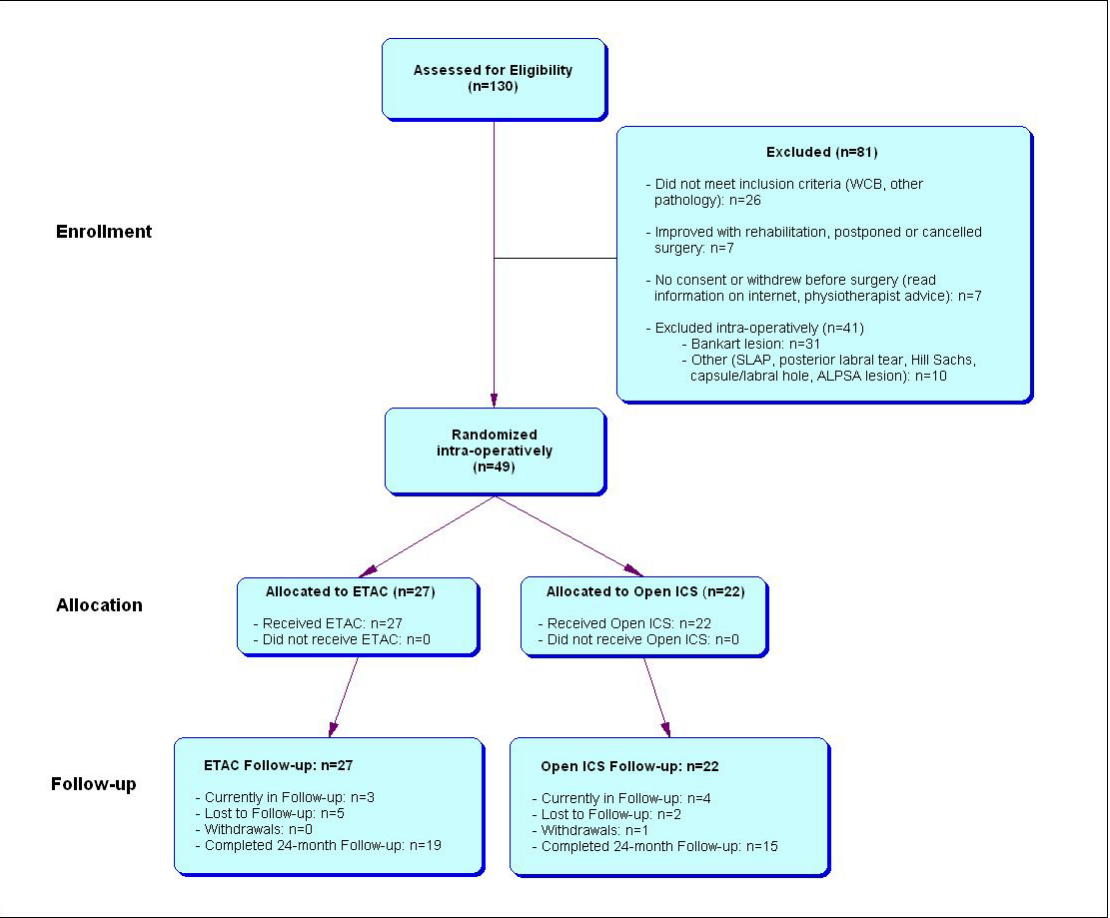
Flow diagram of the recruitment, allocation and follow-up process for the ETAC vs. Open ICS trial between 1999–2005.

Secondly, the initial non-surgical rehabilitative approach for treatment of MDI and MDL-AII patients has proved successful in some cases. This observation combined with patients who have withdrawn from the trial or who have refused surgery has negatively impacted the trial enrollment rate. As shown in Figure 1, at least 7 eligible patients did not undergo surgery because of improvement with rehabilitation, canceling or postponing surgery. Another 7 eligible patients refused research consent or withdrew from the trial pre-operatively. Reasons for withdrawal were based on information found over the internet or from a physiotherapist.

Thirdly, the strict eligibility criteria have led to a high intra-operative exclusion rate. The pre-operative clinical diagnosis of MDI and MDL-AII, mutually exclusive of other types of shoulder instability, is not definitive and requires further confirmation by diagnostic arthroscopy [71]. The presence of a Bankart lesion has been the main reason for intra-operative exclusion. To illustrate this high exclusion rate, of the 90 patients across Canada who have consented to the trial and surgery, 41 (46%) have been excluded intra-operatively, with 31 (76%) of these cases attributed to the presence of a Bankart lesion (Figure 1). Other reasons for intra-operative exclusion are SLAP, Hill Sachs or ALPSA lesions, posterior labral tears and capsular or labral holes (Figure 1). The strict criteria to exclude additional pathologies that contribute to the shoulder instability have consequently resulted in a slow enrollment rate for the trial.

### Safety considerations of open ICS and ETAC

Serious adverse events are reported to the trial's Data Safety Monitoring Committee (DSMC), where the committee is blinded to the treatment allocation. The most common potential complication with open ICS and ETAC surgery is a stiff shoulder, or adhesive capsulitis. This complication can be observed at similar rates with various types of surgical shoulder procedures. In this trial to date, two cases of adhesive capsulitis have been observed. However, the DSMC determined that the complications did not occur with a greater incidence in one intervention compared to the other. The Trial Steering Committee agreed that the events were not serious enough to warrant stopping the trial.

Transient sensory abnormalities, which are common events with shoulder procedures and usually resolve, have not been observed in this trial to date. There is one potential complication that is unique to the thermal procedure, which is catastrophic capsular loss secondary to capsule dissolution. Capsule dissolution has not been observed in either the pilot trial or in this randomized clinical trial.

### Significance of the trial

Shoulder surgeons worldwide have questioned the application of radio-frequency thermal energy in the surgical treatment of shoulder instability. The evaluation of the ETAC procedure in this trial will result in a much clearer understanding of its effectiveness and safety for the treatment of patients with MDI or MDL-AII. It is anticipated that the results of this trial will establish whether or not this technique should be recommended for future clinical use, or abandoned in favor of open procedures.

## Conclusion

This trial was conceived at a time when Electrothermal Arthroscopic Capsulorrhaphy was being performed in the tens of thousands per year in North America. This technique was never tested appropriately in a clinical setting. It was only reported anecdotally and at best, in a prospective case series. Subsequent adverse reports have resulted in a complete turn-around in the enthusiasm of surgeons to use this technique. The current trial has had recruitment issues as a direct result of this anecdotal information. However, there have not been any serious adverse events to compromise the validity of completing the trial and a number of lessons have been learned from this study so far. These lessons include a much better understanding of patient recruitment, a greater appreciation of how to define this population and the value of completing the trial in order to establish higher quality evidence.

## Competing interests

The ETAC probes (Oratec Vulcan Generator electro-thermal system) used in this trial are donated by Oratec Interventions Inc., Menlo Park, CA, USA. NM has received outside funding from Oratec and Smith & Nephew USA to support the annual collaborator meetings for the trial. Absolutely none of this industry support has been used to conduct the trial in any fashion. Peer review funding was obtained initially from The Arthritis Society and subsequently from the Canadian Institutes for Health Research to provide all resources to carry out the multi-centre trial.

## Authors' contributions

NM, RH, TS, AK (deceased) and GF formed the original study team that developed the research question, wrote the study protocol obtained local ethics approval, obtained grant funding and implemented this study. NM, PC and DC drafted and revised this article. RH and GF revised this article.
